# Case Report: Early-onset euglycemic diabetic ketoacidosis following henagliflozin initiation in a treatment-naive patient

**DOI:** 10.3389/fendo.2026.1882272

**Published:** 2026-07-07

**Authors:** Jing Cai, Tiantian Fu, Cuixia Tan

**Affiliations:** Department of Endocrinology and Metabolism, Chengdu First People’s Hospital, Chengdu, Sichuan, China

**Keywords:** early-onset adverse drug reaction, euglycemic diabetic ketoacidosis, metformin, SGLT2 inhibitors, type 2 diabetes mellitus

## Abstract

**Background:**

Sodium-glucose cotransporter 2 (SGLT2) inhibitors are associated with euglycemic diabetic ketoacidosis (euDKA), typically occurring after ≥2 weeks of therapy in the setting of intercurrent illness or surgical stress. The risk of early-onset euDKA in treatment-naive patients—those without prior glucose-lowering therapy or established metabolic stability—remains poorly characterized. We describe a case of severe euDKA occurring within 4 days of SGLT2 inhibitor initiation, precipitated solely by metformin-induced gastrointestinal adverse effects.

**Case presentation:**

A 40-year-old woman with newly diagnosed type 2 diabetes (HbA1c 12.9%) was initiated on metformin, henagliflozin (SGLT2 inhibitor), and chiglitazar. Within 24 hours, she developed nausea and vomiting attributed to metformin intolerance. Despite progressive symptoms and minimal oral intake over 72 hours, she continued all medications without seeking medical care. On day 4, she presented with severe high-anion-gap metabolic acidosis (pH 7.043, bicarbonate 6.5 mmol/L), ketonemia (β-hydroxybutyrate 5.6 mmol/L), and plasma glucose 8.5 mmol/L—consistent with euDKA. Management included intravenous dextrose, insulin infusion, and fluid resuscitation, with resolution by day 3. The patient was discharged on basal insulin, metformin, and chiglitazar (SGLT2 inhibitor discontinued), with no recurrence at 4-week follow-up.

**Conclusion:**

In treatment-naive patients initiating SGLT2 inhibitor-based combination therapy, common metformin-associated gastrointestinal symptoms (10–30% of initiants) can precipitate severe euDKA within days through reduced oral intake and dehydration. Although these are well-recognized precipitants, this case underscores the vulnerability of treatment-naive patients to routine adverse effects during the critical initial phase, highlighting the need for stepwise drug initiation, explicit sick-day education, and scheduled early follow-up.

## Introduction

Diabetic ketoacidosis (DKA) represents one of the most serious acute metabolic complications of diabetes, classically characterized by the triad of hyperglycemia, ketonemia, and metabolic acidosis (1–3). Euglycemic DKA (euDKA), defined by the presence of metabolic acidosis and ketonemia with plasma glucose concentrations <11.1 mmol/L (200 mg/dL), constitutes a diagnostically challenging variant that may delay recognition and treatment, potentially resulting in higher mortality than classic DKA (4–6). Sodium-glucose cotransporter 2 (SGLT2) inhibitors have transformed type 2 diabetes management, with demonstrated benefits in glycemic control, cardiovascular outcomes, and renal protection (7, 8). However, these agents alter normal glucose homeostasis by inducing glucosuria and reducing plasma glucose concentrations, which can mask the hyperglycemic warning signs of DKA (4). Contemporary safety surveillance indicates that SGLT2 inhibitor-associated euDKA predominantly occurs after days to months of therapy, frequently in the context of intercurrent illness, surgical procedures, or markedly reduced oral intake (9–14). Contemporary surveillance indicates that SGLT2 inhibitor–associated euDKA predominantly occurs after days to months of therapy, frequently in the setting of intercurrent illness, surgical procedures, or reduced oral intake (10–14). Although early-onset cases have been documented in treatment-naive patients, these have involved concurrent glucagon-like peptide-1 receptor agonist use, intermittent fasting, or acute cerebrovascular events—multifactorial precipitants that complicate risk attribution (15–17).

We report a case of severe euDKA within 4 days of SGLT2 inhibitor initiation in a treatment-naive patient with newly diagnosed type 2 diabetes, precipitated by metformin-induced gastrointestinal symptoms leading to reduced oral intake and dehydration. Although these precipitants are well-recognized, this case highlights the rapidity of onset and severity of decompensation when routine adverse effects are unrecognized in treatment-naive patients lacking self-management capacity, underscoring the need for enhanced patient education and monitoring during the vulnerable initial weeks of therapy.

## Case presentation

A 40-year-old woman was diagnosed with diabetes mellitus following a routine health examination that revealed a fasting plasma glucose of 17.9 mmol/L (322 mg/dL) and HbA1c of 12.9% (117 mmol/mol). Her body mass index was 28.3 kg/m² (height 168 cm, weight 80 kg). Abdominal ultrasonography demonstrated hepatic steatosis. Lipid profiling revealed mixed dyslipidemia with elevated triglycerides and low-density lipoprotein cholesterol. Autoantibodies associated with autoimmune diabetes (glutamic acid decarboxylase, islet cell antigen, and insulinoma-associated protein-2) were negative, confirming a diagnosis of T2DM. She had no known drug allergies, no family history of diabetes or autoimmune disease, and no history of tobacco or alcohol use. Given her marked hyperglycemia (HbA1c >10%), combination therapy was initiated according to contemporary guideline recommendations: metformin 500 mg twice daily, henagliflozin 5 mg once daily (a selective SGLT2 inhibitor), and chiglitazar 32 mg once daily (a peroxisome proliferator-activated receptor pan-agonist). Standardized diabetes self-management education was provided, including instruction to monitor urinary ketones if symptoms such as nausea or vomiting developed.Serum drug levels of metformin, henagliflozin, or chiglitazar were not measured, as therapeutic drug monitoring is not routinely available or clinically indicated for these agents in standard practice.

Within 24 hours of commencing therapy, the patient developed nausea and recurrent vomiting, which she attributed to dietary indiscretion. Critically, despite progressive symptomatology and declining oral intake, she continued all prescribed antihyperglycemic agents without seeking medical consultation. Over the subsequent 72 hours, oral intake was minimal due to persistent vomiting. On day 4, she developed marked lethargy and was emergently transported to the hospital by family members. Upon presentation to the emergency department, she was somnolent but arousable. Vital signs demonstrated: temperature 36.6 °C, heart rate 108 beats/min, respiratory rate 22 breaths/min, blood pressure 110/70 mmHg, and oxygen saturation 98% on ambient air. Physical examination revealed dry mucous membranes and reduced skin turgor consistent with volume depletion. Laboratory investigations demonstrated: plasma glucose 8.5 mmol/L (153 mg/dL), β-hydroxybutyrate 5.6 mmol/L (reference range: 0.03-0.3 mmol/L), and positive urinary ketones on dipstick analysis. Arterial blood gas analysis confirmed severe high-anion-gap metabolic acidosis: pH 7.043, bicarbonate 6.5 mmol/L, base excess -24.1 mmol/L, and calculated anion gap 28.4 mmol/L (reference range: 10–12 mmol/L). Serum creatinine, amylase, and lactate (2.01 mmol/L) were within normal limits. Computed tomography imaging of the chest, abdomen, and head revealed no acute pathology. Pregnancy testing was negative. There was no history of alcohol consumption or toxin exposure. A diagnosis of euDKA was established based on the characteristic metabolic profile, temporal relationship to drug initiation, and exclusion of alternative etiologies.

## Treatment and clinical course

Management followed the ADA/EASD/JBDS 2024 consensus guideline for hyperglycemic crises (1) and euDKA-specific recommendations from Dhatariya et al. (18). Fluid resuscitation was initiated with isotonic saline (0.9% NaCl) at 1.0 L in the first hour (~15 mL/kg), followed by 250–500 mL/hour titrated to hydration status and hemodynamic response, with an estimated total deficit of 3–4 liters. Concurrently, intravenous 10% dextrose was administered to permit continued insulin infusion without hypoglycemia. Regular insulin was infused at 0.1 units/kg/hour (8 units/hour for this 80-kg patient) without an initial bolus, titrated to maintain plasma glucose 8–12 mmol/L with hourly capillary monitoring. Potassium supplementation (20–30 mEq KCl/L) was added once urine output was confirmed, targeting serum potassium >3.5 mmol/L; levels were monitored every 2–4 hours. Serum phosphate remained within normal limits and did not require supplementation. Bicarbonate was not indicated as pH remained >6.9. Monitoring comprised hourly capillary glucose, β-hydroxybutyrate every 2 hours, venous blood gas and electrolytes every 2–4 hours, and anion gap calculation with each blood gas analysis.

After 24 hours, arterial blood gas analysis demonstrated partial correction of metabolic acidosis (pH 7.223, bicarbonate 12.1 mmol/L). By the third hospital day, ketonemia had resolved (β-hydroxybutyrate <0.3 mmol/L), the anion gap normalized, and the patient successfully transitioned to subcutaneous insulin therapy. Multiple daily injection therapy comprising basal insulin glargine and prandial insulin aspart was introduced following resumption of oral feeding. Oral antihyperglycemic agents—specifically excluding SGLT2 inhibitors—were gradually reintroduced as clinically tolerated. Prandial insulin was subsequently tapered as glycemic stability was achieved.

The patient was discharged on hospital day 8 on the following regimen: insulin glargine 20 units at bedtime (0.25 units/kg), metformin 500 mg twice daily, and chiglitazar 32 mg once daily. Comprehensive discharge education emphasized recognition of DKA warning symptoms and the imperative to withhold SGLT2 inhibitors during intercurrent illness or reduced oral intake. Serial laboratory findings during the clinical course are presented in **Table 1**.

**Table 1:** Patient characteristics across different treatment phases.

Laboratory parameters	At diabetes diagnosis	Initial labs after admission	After 24 hours of treatment	After 72 hours of treatment	Before discharge
HbA1c (%, mmol/mol)	12.92, 117.5	/	/	/	/
Body weight (kg)	80	79	/	/	78
BMI (kg/m2)	28.3	28.0	/	/	27.6
Glucose level (mmol/l)	17.9	8.5	6.7	5.9	6.1
Beta-hydroxybutyrate (mmol/l)	/	5.6	4.8	0.3	0.1
Urine ketones	-	++	+	-	-
Creatinine (mmol/l)	62.3	67.5	/	58.2	53.9
CRP (mg/l)	8.95	12.91	7.82	9.84	6.73
Amylase (U/L)	/	37	29	/	/
ALT (U/L)	52	50	/	51	38
AST (U/L)	25	36	/	34	29
Potassium (mmol/l)	4.02	4.66	3.82	3.95	4.05
Arterial pH	7.396	7.043	7.223	7.356	7.421
BE (mmol/l)	-1.1	-24.1	-16.3	-7.4	1.2
pCO2 (mmHg)	38.5	24.4	23.16	30.72	40.42
Lactic acid level (mmol/l)	1.87	2.01	1.23	1.69	2.01
Anion gap (mmol/l)	12.1	28.4	17.5	13.6	12.7
HCO3 (mmol/l)	24.8	6.5	12.1	16.8	24.9

BMI, Body Mass Index; CRP, C-reactive protein; ALT, alanine aminotransferase; AST, aspartate aminotransferase; BE, base excess.

**Figure 1:**
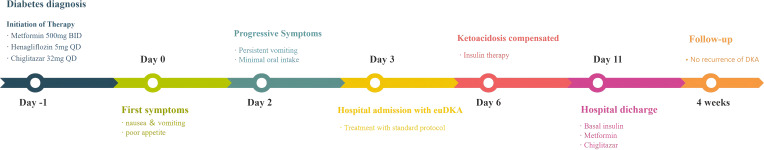
Timeline of the disease course.

At 4-week outpatient follow-up, the patient reported complete resolution of gastrointestinal symptoms with no recurrence of DKA. Continuous glucose monitoring data from the preceding 14 days demonstrated: mean glucose 7.8 mmol/L (140 mg/dL), glucose management indicator 6.7% (50 mmol/mol), coefficient of variation 25%, and 95% time-in-range (3.9-10.0 mmol/L; 70–180 mg/dL), with no hypoglycemic events (<3.9 mmol/L). Body weight had decreased by 2 kg from baseline. Based on these favorable metrics, basal insulin glargine was reduced to 16–18 units at bedtime, with continuation of metformin and chiglitazar. The patient was scheduled for follow-up in 3 months. The timeline of the case is presented in **Figure 1**.

SGLT2 inhibitor rechallenge was not pursued, given the rapid-onset severe euDKA; the patient concurred after shared decision-making. She was maintained on insulin glargine (titrated via CGM), metformin, and chiglitazar, with planned HbA1c monitoring every 3 months and annual cardiovascular and renal risk assessment. Despite persistent anxiety about the DKA episode, she demonstrated excellent adherence and accurate recall of sick-day rules at follow-up.

## Discussion

We report a case of severe euDKA occurring within 4 days of initiating combination therapy with an SGLT2 inhibitor in a treatment-naive patient with newly diagnosed T2DM. The rapid onset and severity highlight the vulnerability of treatment-naive patients during the initial phase of combination therapy. Although the pathophysiological mechanisms—pancreatic hormonal dysregulation, hepatic ketogenesis, and volume contraction—are established, the convergence of metformin-induced gastrointestinal toxicity, severe baseline hyperglycemia, and absence of self-management experience illustrates a clinically significant scenario warranting enhanced vigilance and patient education.

Although SGLT2 inhibitor-associated euDKA typically manifests after ≥2 weeks of therapy, early-onset cases (within 14 days) have been increasingly recognized—precipitated by acute cerebrovascular events (15), combined GLP-1 RA therapy with intermittent fasting (16), or GLP-1 RA-induced vomiting with insulin omission (17) (**Table 2**). Notably, these reported cases all involved major physiological stressors or combined pharmacologic challenges that complicate risk attribution. By contrast, our case was triggered solely by metformin-induced gastrointestinal toxicity—a common adverse effect occurring in 10–30% of initiants (19)—without concurrent insulin use, GLP-1 RA exposure, or self-imposed caloric restriction. This distinction is clinically significant: the absence of confounding factors simplifies attribution to the interaction between SGLT2 inhibitor metabolic effects and metformin-induced reduced intake, underscoring that routine adverse drug effects alone can precipitate severe ketotic decompensation within days of initiation. Moreover, this scenario is more readily encountered yet easily overlooked in practice, as clinicians may not associate common adverse effects with life-threatening metabolic decompensation.

**Table 2:** Comparison of early-onset SGLT2 inhibitor–associated euglycemic diabetic ketoacidosis cases.

Case	Reference	Patient	SGLT2i (Onset)	Time to Onset	Precipitating Factor	Glucose (mg/dL)	pH/HCO_3_^-^ (mmol/L)	β-OHB (mmol/L)	Severity (ICU)	Treatment	Outcome
1	Razok et al. 2022 (15)	45-year-old woman T2DM, Breast cancer	Empagliflozin (Sitagliptin/ metformin, insulin largine)	4 days	Acute ischemic stroke	185	6.90/3	>9.60	Severe (Yes)	Insulin infusion; IV fluids; ICU monitoring	Recovered at 48h; Transferred to rehabilitation
2	Lyu 2025 (16)	38-year-old woman New-onset T2DM	Enavogliflozin (Metformin, semaglutide)	5 days	Intermittent fasting, low-carb diet, GLP-1RA– induced vomiting	137	7.269/11.6	NM; urine ketones (+++)	Moderate (No)	Insulin; discontinued both SGLT2i and GLP-1RA	Resolved by day 3; no recurrence at 3 months;
3	Gandecka- Pempera et al. 2025 (17)	36-year-old man New-onset T2DM, HTN, HTG, Fatty liver	Dapagliflozin (Metformin, semaglutide, insulin lispro)	6 days	GLP-1RA–induced persistent vomiting; insulin omission	182	7.066/7	5.1	Critical (Yes)	Standard DKA protocol refractory; CVVHDF for 48 h	Full recovery; discharged on MDI; TIR 71% at 6 weeks
4	Present Case	40-year-old woman New-onset T2DM	Henagliflozin (Metformin, chiglitazar)	4 days	Metformin induced vomiting, low-carb diet	153	7.043/6.5	5.6	Moderate (No)	Insulin infusion; IV fluids	Resolved by day 3; no recurrence at 4 weeks

SGLT2i, sodium–glucose cotransporter-2 inhibitor; β-OHB, β-hydroxybutyrate; ICU, intensive care unit; T2DM, type 2 diabetes mellitus; GLP-1RA, glucagon-like peptide-1 receptor agonist; NM, not measured; DPP-4i, dipeptidyl peptidase-4 inhibito; HTG, hypertriglyceridemia; HTN, hypertension; CVVHDF, continuous veno-venous hemodiafiltration; MDI, multiple daily injection; NR, not reported; TIR, time in range.

## Pathophysiological considerations

Metformin-induced gastrointestinal toxicity and SGLT2 inhibitor-mediated metabolic effects converged to create a “perfect storm” for euDKA, with synergistic perturbations across hormonal, hepatic, and volume homeostatic axes.

### Pancreatic hormonal dysregulation

SGLT2 inhibitors reduce insulin secretion from β-cells while increasing glucagon secretion from α-cells via KATP channel activation, yielding a higher glucagon-to-insulin ratio that favors hepatic ketogenesis (20–22). Severe baseline hyperglycemia (HbA1c 12.9%) indicated profound β-cell dysfunction and relative insulinopenia at diagnosis (23), rendering the patient particularly vulnerable to SGLT2 inhibition—effects further amplified by metformin-induced fasting.

### Hepatic ketogenesis and volume contraction

Reduced insulin triggers adipose lipolysis and increased free fatty acid delivery to the liver (20). Concurrently, decreased insulin and increased glucagon suppress hepatic acetyl-CoA carboxylase, reducing malonyl-CoA and derepressing carnitine palmitoyltransferase-I (CPT-I)—the rate-limiting enzyme for mitochondrial β-oxidation (24, 25). Accelerated β-oxidation shunts acetyl-CoA into ketone bodies rather than the tricarboxylic acid cycle, particularly when oxaloacetate is diverted to gluconeogenesis (21). Simultaneously, SGLT2 inhibitor-induced osmotic diuresis and natriuresis produced negative volume status (20, 26); superimposed on 72 hours of minimal oral intake from metformin-induced vomiting, this created a “double hit” of volume depletion. Hypovolemia activates counter-regulatory hormones that further stimulate lipolysis and ketogenesis while inducing peripheral insulin resistance (20). Tachycardia, dry mucous membranes, and reduced skin turgor at presentation were consistent with this volume-contracted, ketogenic state.

### The euDKA paradox and treatment-naive vulnerability

In classic DKA, absolute insulin deficiency drives unrestrained hepatic glucose production, yielding marked hyperglycemia. In SGLT2 inhibitor-associated euDKA, the drug’s insulin-independent glucosuric effect creates a continuous “sink” for glucose elimination, maintaining normoglycemia despite severe ketogenesis (20, 21). Despite profound metabolic decompensation (pH 7.043, anion gap 28.4 mmol/L), plasma glucose was only 8.5 mmol/L—reflecting the dominance of glucosuria over endogenous glucose production. This masking effect is particularly perilous in treatment-naive patients lacking the experiential framework to recognize that normoglycemia does not exclude life-threatening ketosis. In newly diagnosed patients with HbA1c >10%, chronic hyperglycemia induces glucotoxicity-mediated β-cell dysfunction and downregulation of SGLT2 expression in pancreatic islets, potentially amplifying the α-cell glucagon response to SGLT2 inhibition (22). Moreover, treatment-naive individuals lack self-management experience to interpret warning signs or implement sick-day rules (27, 28). Our patient’s continued medication adherence despite progressive vomiting—a critical gap in patient education—transformed a common adverse effect into a life-threatening emergency. Treatment-experienced patients, by contrast, possess prior knowledge of adverse effect recognition and sick-day protocols that could prompt earlier intervention.

### Limitations and alternative considerations

We acknowledge several limitations. First, we cannot exclude a contributory role of chiglita-zar, a PPAR pan-agonist that enhances fatty acid ox5idation via PPAR-α activation and can theoretically increase ketogenesis during metabolic stress (29, 30). However, clinical trials demonstrate a favorable safety profile with no reported increase in DKA risk (31), and the t-emporal relationship favors metformin-induced vomiting as the proximate precipitant (symptoms began within 24 hours of metformin initiation). Second, metformin-associated gastrointestinal symptoms are common, and attributing these to euDKA based on a single case should be interpreted with caution. This association remains hypothetical; rather, metformin-induced gastrointestinal toxicity served as the initiating event for reduced oral intake and dehydration, established precipitants of euDKA, in a vulnerable treatment-naive patient with severe baseline hyperglycemia and relative insulinopenia.

### Clinical implications

Current ADA and Chinese Diabetes Society guidelines recommend early combination therapy—including SGLT2 inhibitors or glucagon-like peptide-1 receptor agonists—for patients with newly diagnosed T2DM and severe hyperglycemia (HbA1c >8.5% or >69 mmol/mol) or high cardiovascular risk (32, 33). Our patient met these criteria, yet this case illustrates that guideline-concordant care must be balanced against individual risk profiles, particularly during the vulnerable initial weeks of therapy.

In clinically stable, treatment-experienced cohorts, SGLT2 inhibitor–associated euglycemic diabetic ketoacidosis has been documented at rates of 0.16–0.76 events per 1000 patient-years in post-marketing surveillance data (21, 26). However, the risk in treatment-naive patients during the first week of therapy remains undefined and may be substantially higher, given the absence of established metabolic stability and the potential for early adverse drug interactions. Emerging reports, including the present case, suggest that conventional risk windows may not apply to patients experiencing acute metabolic stressors at diabetes onset.

Based on these observations, we propose the following risk mitigation strategies for clinicians initiating SGLT2 inhibitors in treatment-naive patients (1): stepwise metformin initiation at low dose with gradual titration, assessing tolerability before adding SGLT2 inhibitors (2); explicit written education on warning symptoms, prompt provider contact, SGLT2 inhibitor discontinuation during illness regardless of glucose, and euDKA without hyperglycemia (3); scheduled contact within 1–2 weeks; and (4) individualized risk assessment for gastrointestinal intolerance or reduced intake susceptibility, with consideration of alternative agents or delayed SGLT2 inhibitor initiation.

## Conclusion

This case demonstrates that severe euDKA can occur within days of SGLT2 inhibitor initiation in treatment-naive patients with newly diagnosed T2DM, precipitated by reduced oral intake and dehydration initiated by routine gastrointestinal adverse effects. Although the pathophysiological framework is established, this case underscores the vulnerability of treatment-naive patients during the critical initial phase, when common adverse effects may be unrecognized and self-management capacity is limited. These findings highlight the importance of stepwise drug initiation, explicit sick-day education, and scheduled early follow-up to prevent life-threatening metabolic decompensation.

## Patient perspective

The patient expressed surprise that routine GI symptoms could lead to severe metabolic complications, emphasized the importance of clear patient education regarding SGLT2 inhibitor risks, and provided written informed consent for publication.

## Data Availability

The original contributions presented in the study are included in the article/supplementary material. Further inquiries can be directed to the corresponding author.
